# A highly divergent Encephalomyocarditis virus isolated from nonhuman primates in Singapore

**DOI:** 10.1186/1743-422X-10-248

**Published:** 2013-08-02

**Authors:** Dawn Su-Yin Yeo, Jing Er Lian, Charlene J Fernandez, Yueh-Nuo Lin, Jasper Chin-Wen Liaw, Moi-Lien Soh, Elizabeth Ai-Sim Lim, Kwai-Peng Chan, Mah-Lee Ng, Hwee-Cheng Tan, Serena Oh, Eng-Eong Ooi, Boon-Huan Tan

**Affiliations:** 1 Defence Medical and Environmental Research Institute, DSO National Laboratories, #13-00, 27 Medical Drive, Buona 117510, Singapore; 2 School of Biological Sciences, Nanyang Technological University, Nanyang, Singapore; 3 Agri-Food and Veterinary Authority of Singapore, Singapore, Singapore; 4 Department of Pathology, Singapore General Hospital, Changi, Singapore; 5 Department of Microbiology, National University of Singapore, Singapore, Singapore; 6 Duke-NUS GMS, National University of Singapore, Singapore, Singapore; 7 Wildlife Reserves Singapore, Singapore, Singapore; 8 Saw Swee Hock School of Public Health, National University of Singapore, Singapore, Singapore; 9 Department of Civil and Environmental Engineering, National University of Singapore, Singapore, Singapore

**Keywords:** Encephalomyocarditis virus, Variant virus, Fatal acute myocarditis, Orang utan

## Abstract

**Background:**

In 2001 and 2002, fatal myocarditis resulted in the sudden deaths of four, two adult and two juvenile, orang utans out of a cohort of 26 in the Singapore Zoological Gardens.

**Methods:**

Of the four orang utans that underwent post-mortem examination, virus isolation was performed from the tissue homogenates of the heart and lung obtained from the two juvenile orang utans in Vero cell cultures. The tissue culture fluid was examined using electron microscopy. Reverse transcription and polymerase chain reaction with Encephalomyocarditis virus (EMCV)-specific primers targeting the gene regions of VP3/VP1 and 3D polymerase (3Dpol) confirmed the virus genus and species. The two EMCV isolates were sequenced and phylogenetic analyses of the virus genes performed. Serological testing on other animal species in the Singapore Zoological Gardens was also conducted.

**Results:**

Electron microscopy of the two EMCV isolates, designated Sing-M100-02 and Sing-M105-02, revealed spherical viral particles of about 20 to 30 nm, consistent with the size and morphology of members belonging to the family *Picornaviridae*. In addition, infected-Vero cells showed positive immunoflorescence staining with antiserum to EMCV. Sequencing of the viral genome showed that the two EMCV isolates were 99.9% identical at the nucleotide level, indicating a similar source of origin. When compared with existing EMCV sequences in the VP1 and 3Dpol gene regions, the nucleotide divergence were at a maximum of 38.8% and 23.6% respectively, while the amino acid divergence were at a maximum of 33.9% and 11.3% respectively. Phylogenetic analyses of VP1 and 3Dpol genes further grouped the Sing-M100-02 and Sing-M105-02 isolates to themselves, away from existing EMCV lineages. This strongly suggested that Sing-M100-02 and Sing-M105-02 isolates are highly divergent variants of EMCV. Apart from the two deceased orang utans, a serological survey conducted among other zoo animals showed that a number of other animal species had neutralizing antibodies to Sing-M105-02 isolate, indicating that the EMCV variant has a relatively wide host range.

**Conclusions:**

The etiological agent responsible for the fatal myocarditis cases among two of the four orang utans in the Singapore Zoological Gardens was a highly divergent variant of EMCV. This is the first report of an EMCV infection in Singapore and South East Asia.

## Introduction

Between July 2001 and January 2002, four (3 Bornean, *Pongo pygmaeus* and 1 Sumatran, *Pongo abelii*) orang utans, two (male and female) adults and two (male and female) juveniles, from a cohort of 26 in the Singapore Zoological Gardens died suddenly. Post-mortem examination of the four orang utans revealed pathology characterized by multifocal myocarditis, pulmonary congestion and edema, hydropericardium, hydrothorax and Ascites.

A literature review revealed several reports describing Encephalomyocarditis virus (EMCV) as the etiological agent of viral myocarditis in captive primates. These primates included semi-wild bonobos [1], baboons [2], chimpanzees [3], lemurs [4], rhesus macques [5], and orang utans [3,6]. EMCV infection was also implicated in the deaths of various animals in Audubon Park Zoo, New Orleans, in 1985 [7]; and in elephants in a Florida zoo in 1997 [8]. Outside the United States, sporadic outbreaks of EMCV involving a variety of zoo animals had occurred in Taronga Zoo in Australia from 1987 to 1995 [3]; in free-living elephants in Kruger National Park, South Africa between 1993 and 1994 [9,10]; and in an Italian zoo affecting 15 different primates between 2006 and 2008 [4]. In addition, EMCV infections were reported in Russia from monkeys bred from the Sukhumi Breeding Center in 1974; and in the Adler Breeding Center, since 2001 [11]. EMCV has also been recognized as a porcine pathogen, with EMCV infections in European pigs associated with sudden deaths and reproductive failure [12-16]. In Asia, EMCV was isolated from pigs in South Korea and implicated as the cause of reproductive failure in pigs in Taiwan [17,18]. Apart from EMCV, there has been one other report describing Coxsackie virus B4 as the etiological cause of fatal myocarditis in a female orang utan at the Okinawan Zoo in 1999 [19].

Although the potential for EMCV to cross the species barrier has been demonstrated as seen from the various zoo outbreaks described above, human cases have fortunately been rare. Sporadic human EMCV infections and disease have been documented by virus isolation from different specimen types such as serum, stool samples, cerebrospinal fluid and throat washings [20,21]. A recent study describing the etiology of acute febrile disease in locations across South America concluded that there is evidence supporting a role for EMCV in human infection and febrile illness [22]. However, the extent of the effect of EMCV on human health is still largely unknown because the disease is so infrequent in humans.

We hypothesized that the etiological agent behind the deaths of the orang utans in the Singapore Zoological Gardens was either a Coxsackie virus B, or more likely, an EMCV. Although both viruses are members of the same family, *Picornaviridae*[23], they belong to different genera as Coxsackie virus B is an *Enterovirus*, and EMCV, a *Cardiovirus*. The *Picornaviridae* family is one of the largest and most diverse families of RNA viruses, and includes etiological agents that are responsible for a wide variety of human and animal diseases [23].

The picornavirions are small, non-enveloped and spherical, with a diameter of about 20 to 30 nm. Picornaviruses have a single-stranded, positive sense RNA genome that is between 7.2 and 9 kb in length and contain a large opening reading frame (ORF). The ORF encodes for a polyprotein that comprises both non-structural and structural elements divided into three primary precursor molecules, namely P1, P2 and P3, encoding for 11 distinct proteins. The structural proteins VP4, VP2, VP3 and VP1 make up the viral capsid and are encoded in the P1 region towards the 5′-end of the genome. Non-structural proteins are derived from the P2 and P3 regions and are encoded towards the 3′-end of the genome, the largest of which is the RNA-dependent RNA polymerase (3Dpol). In addition, cardioviruses code for an L (Leader) protein at the N-terminus of their polyproteins. The genomic RNA also contains a highly structured 5′-UTR (untranslated region) that includes an internal ribosome entry site (IRES) from which viral protein translation is initiated in a cap-independent manner. The shorter 3′-UTR terminates with a heterogeneous poly(A) tail that is known to be involved in the binding process of the viral replicase complex.

In this paper, we described the cell culture isolation and identification of two viral isolates obtained post-mortem from the two juvenile Bornean orang utans. The viral genomes of the two isolates were >95% sequenced, with the complete genome sequence lacking about 200 nucleotides (nt) from the 5′ end. Phylogenetic and sequence analyses suggested that the newly isolated viruses are highly divergent variants of EMCV and possibly a new serotype of the virus. This is the first report of EMCV infection in Singapore and South East Asia and its molecular characterization.

## Results

### Virus isolation and preliminary diagnosis

Virus isolates, designated Sing-M105-02 and Sing-M100-02, were successfully isolated in African Green Monkey kidney (Vero) cells from the heart tissues of the first juvenile orang utan, and lung tissues of the second autopsied juvenile orang utan, respectively. Cytopathic effect (CPE) was observed in Vero cells inoculated with the heart homogenate, at 2 days post-infection (pi). Infected-Vero cells were distorted from its normal shape, became granular and finally rounded-up (results not shown). Similar observations of CPE were made with the inoculation of lung homogenate at 3 days pi. Uninfected Vero cells did not show any signs of CPE. Both virus isolates were also inoculated into Mardin Darby Canine Kidney (MDCK) cells (data not shown). At 1 to 2 days pi, CPE in virus infected-MDCK cells was characterized by rounded and wrinkled appearances.

**Figure 1:**
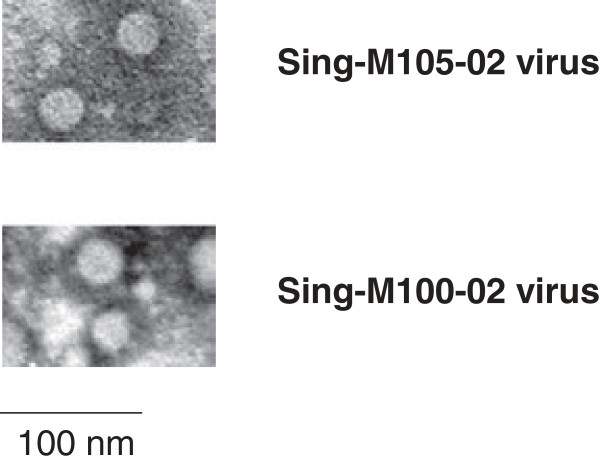
**Electron micrographs showing viruses from the Sing-M105-02 and Sing-M100-02 isolates.** Viruses were concentrated from the tissue culture fluid of virus-infected Vero cells and negatively-stained. Magnification at x50,000. Scale bars represent 100 nm.

The viruses in infected-Vero tissue culture fluid were negatively stained and examined with electron microscopy. The electron micrographs revealed numerous small round virus particles with smooth appearances in the range of 20 to 30 nm for both Sing-M105-02 and Sing-M100-02 isolates (Figure 1). The morphology and size observed are consistent with virus members belonging to the family of *Picornaviridae*[23].

**Figure 2:**
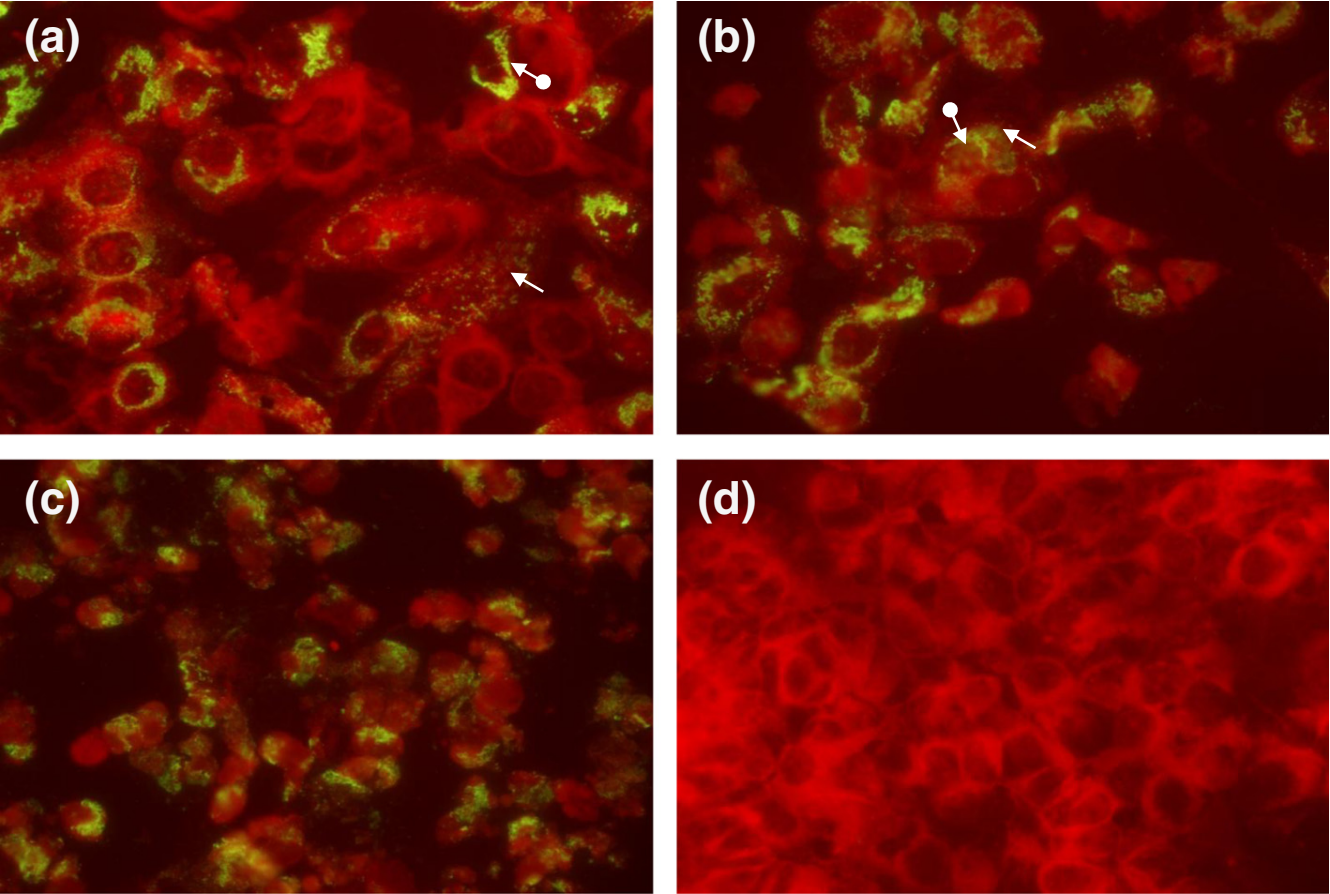
**Immunoflorescence staining of Vero cells infected with (a) Sing-M105-02 isolate, (b) Sing-M100-02 isolate, and (c) EMCV(Aust) strain.** The infected cells were allowed to react with porcine polyclonal antibodies raised against EMCV. The virus-infected Vero cells stained greenish yellow in the cytoplasm (), and at the perinuclear region (), indicating the presence of virus antigens. **(d)** Uninfected Vero cells stained red with counter stain. Magnification at x60.

A second passage of the virus isolates in Vero cells resulted in rapid and advanced CPE a day after inoculation. Sing-M105-02 (Figure 2a) and Sing-M100-02 (Figure 2b) virus-infected Vero cells reacted to polyclonal antiserum raised to EMCV with specific green and yellow fluorescence staining in the cytoplasm. The perinuclear regions were stained more intensely. These results were consistent with observations made for EMCV(Aust)-infected Vero cells (Figure 2c), indicating that the Sing-M105-02 and Sing-M100-02 viruses could be EMCV. Cytoplasmic areas appeared red with counter stain Evans Blue, and no green fluorescence was observed in uninfected Vero cells (Figure 2d).

### Virus identification and full-genome molecular characterization

Viral RNA, extracted from the virus-infected tissue culture fluid, was subjected to RT-PCR analysis using one set of primers that were specific for enterovirus detection [24,25], while another two sets of primers specific for the detection of EMCV were also used [13,26] (Table 1). No products were detected from the enterovirus RT-PCR, while approximately 855 bp and 286 bp products were observed with the VP3/VP1 and 3Dpol primer sets respectively for both Sing-M105-02 and Sing-M100-02 viruses (results not shown). The sizes were consistent with reports using the same primer sets [26-28]. No results were observed on RT-PCR performed on sample processed from uninfected Vero cells or when water was used to replace the viral RNAs. All the PCR assays were repeated at low stringency so as to detect potentially related viruses. The RT-PCR results indicated that Sing-M105-02 and Sing-M100-02 isolates belong to *Cardiovirus* genus and to EMCV species.

**Table 1:** Sequences and references for primers used for RT and PCR reactions

**Virus designation**	**Primer names and sequences (5′ to 3′)**	**Gene target**	**Amplicon size**	**References**
EMCV	P1 = CCC TAC CTC ACG GAA TGG GGC AAA G	3D polymerase gene	286 bp	[13]
	P2 = GGT GAG AGC AAG CCT CGC AAA GAC AG			
EMCV	P9 = ATC AAG ACT CCA GCT CTC GGG GTC A	VP3/VP1 capsid gene	855 bp	[26]
	P10 = TGC CTA TCT CAC CTG CCC CCT GGA G			
Enterovirus	RotbartFor = CCT CCG GCC CCT GAA TGC GGC TAAT	5′ non-coding region	153 bp	[25]
	RotbartRev = ACC GAC GAA TAC CAC TGT TA			
Enterovirus	EV2 = TCC GGC CCC TGA ATG CGG CTA ATC C	5′ non-coding region	113 bp	[24]
	EV1 = ACA CGG ACA CCC AAA GTA GTC GGT CC			

We managed to sequence more than 95% of the full virus genome (i.e. ~7.6 kb) of both Sing-M105-02 and Sing-M100-02 isolates by primer walking (Table 2). This included the entire ORF and 3′-UTR of the virus. Despite numerous attempts to amplify out the 5′-UTR region, we were unable to obtain about 200 nt of virus sequence from the 5′ end of the genome, including the poly(C) tract. This difficulty in sequencing the 5′-UTR region was also reported for another EMCV strain [29], possibly due to the high GC content and complex secondary structure in this region. The Sing-M105-02 and Sing-M100-02 isolates showed a high sequence identity of 99.9% in the entire ORF (Tables 3 and 4), indicating that both viruses represent separate isolates of a single virus strain, probably originating from a similar source.

**Table 2:** Primers used for RT-PCR and sequencing of Sing-M105-02 and Sing-M100-02 virus isolates

**Primer designation**	**Sequence (5′ to 3′)**	**Nucleotide position**	**Forward/Reverse**	**Primer reference**
		**(estimated)**		
EMCVF445	TTTGCAGGCAGCGGAATC	490	Forward	This study
M105R524	CGTGCCGCCTTTGCAGGTGTCTG	524	Reverse	This study
M105R870	CCATTTCTGTATTGCAGGGCAGAGC	870	Reverse	This study
EMCVF1070	TAATGCAGCCGGGTCTGACC	1070	Forward	This study
M105R2369	CAAAATAACTGAGTTTGGAC	2369	Reverse	This study
EMCV2737R	GCTACAAAATCTGGAGTAGCA	2689	Reverse	This study
EMCV-P10	TGCCTATCTCACCTGCCCCCTGGAG	2674	Forward	[26]
M105R2843	ACTGTGGTCCTGGTGTCA	2800	Reverse	This study
M105VP1F419	AATCATGGCTTAGCTG	3000	Forward	This study
M105VP1F540	CCGGAACCTCAAACCA	3200	Forward	This study
M105VIPR768	ACAGTAGGTCTCGGACA	3354	Reverse	This study
EMCV-P9	ATCAAGACTCCAGCTCTCGGGGTCA	3529	Reverse	[26]
M105Middle-F505	AACCCGTGGAAGAGAACCT	3710	Forward	This study
EMC-2B65R	TCGGCAGTAGGGTTTGAG	3975	Reverse	[26]
M105F4204	ATTGCAGGAATGACAATT	4200	Forward	This study
M105Contiq3-R335	AGTTGCAGGGTTTTTGGTG	5500	Reverse	This study
M105Contiq3-F327	CCTGCAACTGCTGGATGT	5839	Forward	This study
M105F5906	ACAGGAAAAGATACCGATGT	5980	Forward	This study
M105R6000	TAATGGTCCTGAATTAAG	6000	Reverse	This study
M105R6500	ATCGAATTTAGACAACACTGC	6500	Reverse	This study
M105POLF16	ACACTAGATGATGTAGTTT	7000	Forward	This study
M105POLR158	GTCACTGAGGTGAGTT	7460	Reverse	This study
EMCV-P1	CCCTACCTCACGGAATGGGGCAAAG	7631	Reverse	[13]
EMCV-P2	GGTGAGAGCAAGCCTCGCAAAGACAG	7370	Forward	[13]

**Table 3:** **Percent nucleotide (nt) and deduced amino acid (aa) identities of the entire ORF, VP1 and 3Dpol genes of Sing-M105-02 compared to Sing-M100-02, other EMCV and The ilovirus strains (all members of the *Cardiovirus *genus)**

		**ORF**		**VP1**		**3Dpol**	
	**Virus strain**	**nt**	**aa**	**nt**	**aa**	**nt**	**aa**
**EMCV**	Sing-M100-02	99.9	99.9	99.9	99.6	100.0	100.0
	EMCV D (M22458)	75.2	86.3	73.6	86.3	78.4	88.9
	EMCV B (M22457)	75.1	86.3	73.5	86.3	78.4	89.8
	EMCV D (M37588)	75.3	86.4	73.5	86.3	78.6	89.8
	EMCV PV2 (X87335)	75.3	86.4	73.4	85.9	78.6	89.8
	EMCV G424-90 (AJ617362)	NA		73.3	85.2	NA	
	EMCV pEC9 (DQ288856)	75.5	86.6	73.3	87.0	79.2	88.9
	EMCV BEL-288791 (AF356822)	75.6	86.7	73.2	86.6	79.2	89.8
	EMCV CBNU (DQ517424)	75.6	86.7	73.2	86.3	79.2	88.7
	EMCV GX0601 (FJ604852)	75.5	86.6	73.2	86.6	79.1	89.6
	EMCV Mengo M (L22089)	75.5	86.6	73.2	87.0	78.6	88.7
	EMCV Mengo Rz-pMwt (DQ294633)	75.5	86.6	73.2	87.0	78.6	89.8
	EMCV pV21 (X74312)	75.5	86.5	73.2	86.3	79.3	89.6
	EMCV GX0602 (FJ604853)	75.4	86.4	73.0	86.6	78.9	89.6
	EMCV HB1 (DQ464063)	75.5	86.6	73.0	86.6	79.2	89.8
	EMCV K11 (EU780149)	75.5	86.6	73.0	86.3	79.2	89.3
	EMCV K3 (EU780148)	75.5	86.3	73.0	85.9	79.1	88.7
	EMCV BJC3 (DQ464062)	75.5	86.6	72.9	86.3	79.2	89.8
	EMCV GXLC (FJ897755)	75.5	86.4	72.9	85.2	79.0	89.8
	EMCV R (M81861)	75.4	86.2	72.8	85.6	79.1	90.2
	EMCV Ruckert (M81861)	75.4	86.2	72.8	85.6	79.1	89.6
	EMCV MN-30 (AY296731)	75.1	86.4	72.4	86.6	78.6	89.8
	**EMCV 1086C (DQ835185)**	74.6	86.9	71.8	87.0	**76.4**	**88.7**
	EMCV C108-95 (AJ617359)	NA		71.1	87.0	NA	
	**EMCV RD1338 (JX257003)**	**73.6**	**82.5**	**61.2**	**66.1**	80.9	89.6
**Theilovirus**	SAFV-1 (EF165067).seq	56.7	53.6	77.4	88.6	60.8	53.5
	SAFV-2 (AM922293).seq	57.1	54.0	75.0	85.9	57.5	52.8
	TMEV BeAn 8386 (M16020).seq	57.3	54.8	75.3	83.9	58.5	51.0
	TMEV BeAn 8386 S2 (DQ401688).seq	57.3	54.9	75.5	83.9	58.7	51.0
	TMEV DA (M20301).seq	57.1	55.1	75.5	81.8	60.4	50.6
	TMEV GDVII (M20562).seq	57.7	55.0	71.5	78.9	58.1	50.6
	TMEV GDVII (X56019).seq	57.7	55.0	71.5	78.9	57.9	50.2

The virus isolate names are shown followed by the GenBank accession number in brackets. The EMCV strains with the highest divergence from Sing-M105-02 and Sing-M100-02 are in bold.

**Table 4:** Percent nucleotide identity of the L protein, P1 capsid, P2 non-structural, P3 non-structural and 3′UTR regions of Sing-M105-02 compared to Sing-M100-02 and other EMCV strains

**Virus strain**	**Proposed lineage**	**L**	**P1**	**P2**	**P3**	**3′ UTR**
Sing-M100-02	D	100.0	99.9	99.9	100.0	100.0
EMCV Ruckert (M81861)	A	79.1	75.6	74.9	74.8	84.1
EMCV pV21 (X74312)		79.1	75.6	74.7	75.0	85.7
EMCV PV2 (X87335)		80.1	74.9	75.4	74.8	85.6
EMCV B (M22457)		79.1	74.9	75.1	74.7	85.4
EMCV D (M22458)		80.1	75.0	75.1	74.7	85.6
EMCV Mengo M (L22089)	C	81.6	76.1	74.5	74.9	87.9
EMCV 1086C (DQ835185)	B	86.1	75.5	73.1	73.3	NA
EMCV RD1338 (JX257003)	E	80.1	67.7	76.4	77.3	81.2

The proposed EMCV lineage is also indicated. The virus strains names are shown followed by the GenBank accession number in brackets. NA indicates sequence data not available.

### Sequence comparison with existing EMCV strains

To investigate the genetic relationship of Sing-M105-02 and Sing-M100-02 isolates with existing cardioviruses (EMCV and Theiloviruses), we performed sequence comparison for the entire ORF, VP1 capsid and 3Dpol gene regions. Table 3 reflects the percent nt and deduced amino acid (aa) identities of the entire ORF, VP1 capsid and 3Dpol gene regions for Sing-M105-02 and Sing-M100-02 isolates compared with other fully sequenced *Cardiovirus* strains. In the conserved 3Dpol region, the extent of sequence identities between the Sing-M100-02 and Sing-M105-02 isolates and other EMCV strains ranged from 76.4% to 80.9% (nt level) and 79.4% to 90.5% (aa level). The lowest sequence relationships were observed in the variable VP1 capsid region, with percent identities ranging from 61.2% to 73.6% (nt level) and 66.1% to 87.0% (aa level). Table 4 compares the relationship of the Sing-M105-02 and Sing-M100-02 isolates against selected fully sequenced EMCV strains in the different coding regions across the entire ORF (namely L, P1, P2 and P3), and also the 3′-UTR region. We note that nt sequence identities were all below 80% in the P1, P2 and P3 regions between our isolates and existing EMCV strains, once again demonstrating the high divergence of the Sing-M105-02 and Sing-M100-02 isolates.

**Figure 3:**
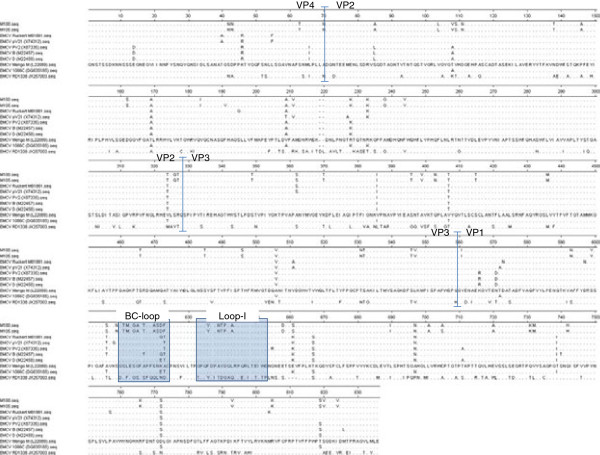
**Alignment profile of the deduced amino acid (aa) sequences of the P1 structural region of Sing-M105-02 and Sing-M100-02 isolates with selected EMCV strains.** The Mengo virus sequence is used as the reference strain to reflect the proposed neutralizing antigenic regions and aa differing from the reference sequence are shown. The amino acids of Sing-M105-02 and Sing-M100-02 isolates differ from the Mengo virus sequence in the putative antigenic BC-loop and loop I regions are highlighted (boxed).

The aa sequence alignment of the P1 structural region was next examined and we observed significant differences in the BC-loop and loop I regions in the VP1 capsid of the Sing-M105-02 and Sing-M100-02 isolates with other EMCV strains (see boxed region in Figure 3). The BC-loop and loop I regions of the VP1 capsid protein are two putative neutralizing antigenic sites proposed in the EMCV Mengo virus strain [30,31]. Taken together, our sequence data indicated that the Sing-M105-02 and Sing-M100-02 isolates have a highly divergent nature compared to any known strains of EMCV.

### Phylogenetic analysis

**Figure 4:**
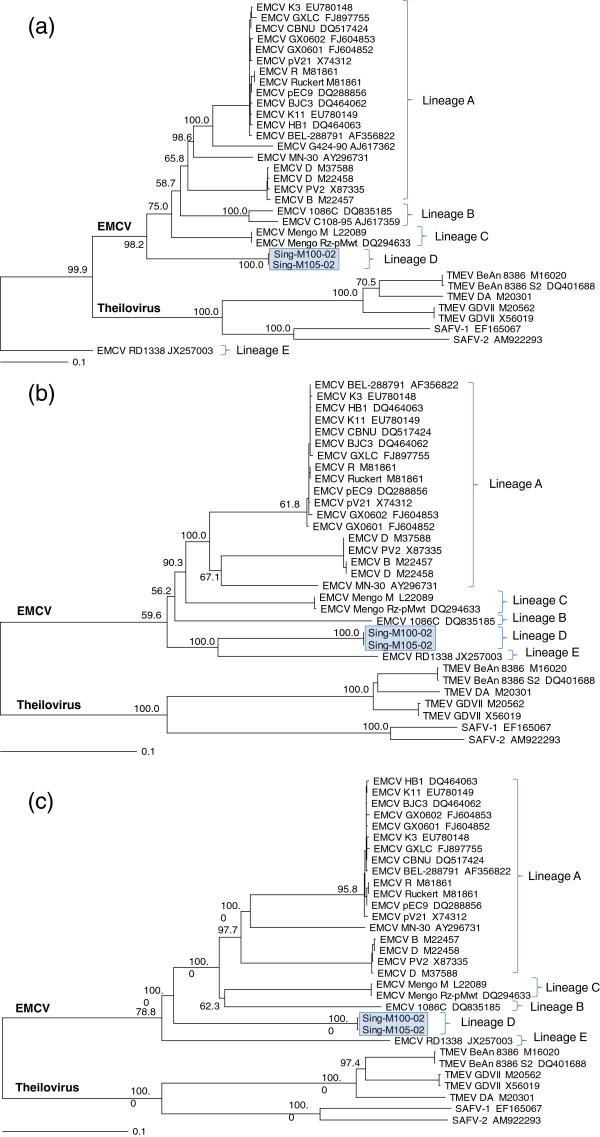
**Phylogenetic trees of the full genes of the (A) VP1 capsid, (B) 3D polymerase (3Dpol), and (C) open reading frame (ORF) of Encephalomyocarditis viruses (EMCV) and Theiloviruses based on their nucleotide sequence.** Isolate names are followed by their GenBank accession number. The EMCV isolates generated in this study, Sing-M100-02 and Sing-M105-02, are highlighted in a shaded box. Percentage bootstrap values (1000 trials) of the major nodes are shown. Trees were constructed from CLUSTAL W method in the program MegAlign (DNASTAR, Lasergene Version 8), and viewed with TreeView 1.6.6.

We included fully sequenced EMCV and Theilovirus strains in our phylogenetic trees to demonstrate the divergence of the 2 species in the genus *Cardiovirus*, as well as to emphasize the diversity and clustering of various EMCV strains (Figure 4). Our phylogenetic analyses showed branching of the EMCV strains into four main lineages, A, B, C and D, at the nt level in the VP1 and 3Dpol regions, as well as for the entire ORF (Figure 4). Lineages A, B and C concurred with similar clusterings of EMCV strains as described previously [10,26,32]. However, the Sing-M105-02 and Sing-M100-02 viruses clustered separately as a distinct group by themselves in lineage D (Figure 4). A recent report of a second serotype of EMCV, isolate RD1338 [29], also clustered by itself in lineage E, highly distinct from the Sing-M105-02 and Sing-M100-02 viruses (lineage D) and other EMCV in lineages A, B and C. Our phylogenetic studies further demonstrated that Sing-M105-02 and Sing-M100 viruses are highly divergent strains of EMCV.

### Serological survey of the zoo animals

In order to investigate whether other zoo animals had been exposed to the EMCV variant, a serological survey was conducted. Sera were collected from a variety of zoo animals, in particular animals residing near the orang utans, and titrated for neutralizing antibodies to both Sing-M105-02 virus and EMCV(Aust). A selection of capybaras (80%), chimpanzees (31%) and orang utans (50%) exhibited neutralizing antibody titers to Sing-M105-02 virus, strongly indicating that they had been exposed to the virus (Table 5). In total, 22 of 122 zoological animals (18%) exhibited neutralizing antibody titers to the Sing-M105-02 isolate. All of the chimpanzees, except one, and an orang utan also had high neutralizing antibody titer to EMCV(Aust) (results not shown). Our serological survey indicated that there may have been two strains of EMCV circulating among the zoo animals. One of the viruses could be an EMCV, closely related to EMCV(Aust), although no attempts at virus isolation were made from these exposed animals. The second virus could be a variant of the EMCV, similar to Sing-M105-02 and Sing-M100-02.

**Table 5:** Prevalence of neutralizing antibodies to the Sing-M105-02 isolate in different animals from the Singapore Zoological Garden

**Animal**	**Number tested**	**Number positive (% positive)**
Agile gibbons	6	0
Cape hunting dog	1	0
Capucin Jito	1	0
Capybaras	10	8 (80)
Chimpanzees	13	4 (31)
Colobus monkeys	3	0
Coloque	1	0
Gibbons	21	1 (5)
Howler monkeys	3	0
Japanese monkeys	2	0
Komodo dragons	2	0
Macaques	19	0
Mandrill	2	0
Orang utans	14	7 (50)
Patas monkey	1	0
Pig tail macaques	2	0
Raccoon	1	0
Rats	2	2 (100)
Lemur	2	0
Siamang	3	0
Slow loris	1	0
Spider monkeys	5	0
White nose gueron	4	0
**Total**	**122**	**22 (18)**

As previous reports have implicated rodents in the initiation of EMCV outbreaks, a rodent control program was initiated at the Singapore Zoological Gardens as a control measure for the management of the myocarditis outbreak [33]. However, only two rats were caught, and these were bled. The two rats were sampled and demonstrated high titers of neutralizing antibodies to Sing-M105-02 virus (Table 5); one of the rats had neutralizing antibody to both Sing-M105-02 virus and EMCV(Aust) (results not shown). Our data provided evidence that the rats were exposed to either the Sing-M105-02 virus, or both the Sing-M105-02 virus and another EMCV.

## Discussion

The virus outbreak in the Singapore Zoological Gardens took place over an estimated period of 8 months, and occurred in orang utans of various ages, as well as sporadically among other zoo animals. This is the first report of an EMCV infection in Singapore. There were altogether four deaths in the orang utan cohort, caused by acute myocarditis. Other animals, two zebras, one spider monkey, one guanaco, and two capybaras also died of similar clinical signs. One adult orang utan displayed respiratory problems but recovered with intensive supportive therapy.

From the two juvenile orang utans that died, viruses were isolated successfully from the heart (designated Sing-M105-02) and lung (designated Sing-M100-02) homogenates in Vero cells. Virus isolation was not performed on the two autopsied adult orang utans. The viral agents isolated in Vero cells were examined under transmission electron microscopy and the viruses showed similar morphology to the picornaviruses (Figure 1). In addition, virus-infected Vero cells showed positive immunofluorescence to polyclonal antiserum raised to EMCV (Figure 2). Our preliminary characterization of the Sing-M105-02 and Sing-M100-02 viruses indicated that the etiological agent in the Singapore zoo outbreak was an EMCV.

EMCV-specific primers targeting the VP3/VP1 capsid and 3Dpol genes were able to PCR-amplify products from the viral isolates. These primer sets had been tested on a wide variety of EMCV isolates from different geographic locations, different host species, and at different times [26-28]. Structural proteins such as the VP1 capsid, have been shown to be suitable for phylogenetic analyses within different genera and species of picornaviruses [24,33-41]. Furthermore, analysis using the VP1 capsid could lead to the discovery of a novel variant such as the discovery reported for Hepatitis A [42]. Analysis at the VP1 capsid region revealed a maximum of 38.8% nt and 33.9% aa divergence between the Sing-M105-02 and Sing-M100-02 isolates and existing EMCV strains (Table 3). In addition, aa alignment of Sing-M105-02 and Sing-M100-02 against other EMCV strains showed considerable variation in two putative neutralization antigenic sites in VP1 (Figure 3), further supporting our hypothesis that these viral isolates can represent a new divergent group of EMCV and possibly a new serotype in the EMCV species.

The picornaviral polymerases or 3Dpol genes, on the other hand, are well conserved during evolution, and have been used as a common marker for comparisons between genera in phylogenetic studies [43-45]. They can also be used to determine the degree of variation between the newly isolated viruses with existing EMCV strains. For example in Foot-and-Mouth-Disease viruses, high sequence divergence found in the 3Dpol regions is an indication of the virus strains diverging from the same isolate [46]. Sequence comparison for Sing-M105-02 and Sing-M100-02 isolates with existing EMCV strains showed a maximum divergence of 24.6% and 11.3% at the nt and aa levels respectively (Table 3), suggesting that they are indeed highly divergent variants of EMCV.

In addition, the phylogenetic analyses based on complete virus gene sequences (as compared to partial gene sequences) afforded us increased confidence that the Sing-M105-02 and Sing-M100-02 isolates constituted a divergent group of EMCV variants, as they clustered distinctly away from the other lineages of EMCV strains (Figure 4). This high degree of divergence within the EMCV species is perhaps not surprisingly as viruses with RNA genomes are generally reported to have a high rate of mutation [47-49].

Our serological survey of the animal population further suggested that there were at least two virus strains circulating in the Singapore Zoo; the first, an EMCV(Aust)-like virus, and the second, a variant of EMCV. High seropositive rates for the EMCV variant was found in capybaras, chimpanzees, orang utans and two rats caught in baited traps. In addition, neutralizing antibodies to EMCV(Aust) were also found in all except one of the chimpanzee, one orang utan and one of the rats, indicating prior exposure to single or dual infections with the viruses. Although no attempt was made to recover the virus from these animals, we postulated that the EMCV(Aust)-like virus could have been the parental virus from which Sing-M105-02 and Sing-M100-02 viruses gradually evolved. Rats have been considered to be the primary host reservoir and disseminators of EMCV, and they have been implicated in other zoological outbreaks of EMCV [2-4,7,9]. The rats could also have initiated the sporadic infections, by spreading both the parental and its variant virus in the zoo. However, this hypothesis remains to be investigated.

## Conclusions

The etiological agent responsible for the outbreak for 2 of the 4 cases of fatal myocarditis in orang utans at the Singapore Zoological Gardens was an EMCV. High divergence in nt sequences of the VP1 capsid and 3Dpol genes as compared to other known EMCV strains, indicated that the outbreak was caused by a highly divergent variant of EMCV and possibly a new serotype of the virus. This is the first report of an EMCV infection in Singapore and South East Asia.

## Materials and methods

### Specimens

Post-mortem heart and lung tissues were obtained from the two juvenile orang utans. In addition, a total of 122 serum samples from various zoo animals were collected for sero-epidemiological study. These consisted of 6 agile gibbons, 1 cape hunting dog, 1 capuchin monkey, 10 capybaras, 13 chimpanzees, 3 colobus monkeys, 1 coloque monkey, 21 gibbons, 3 howler monkeys, 2 Japanese monkeys, 2 komodo dragons, 19 macaques, 2 mandrills, 14 orang utans, 1 patas monkey, 2 pig-tailed macaques, 1 raccoon, 2 lemurs, 3 siamangs, 1 slow loris, 5 spider monkeys and 4 white nose guenons. Two rats were also captured in a baited trap.

### Virus isolation and propagation

Heart and lung tissues were collected from the post-mortem analyses of two juvenile orang utans. The tissues were homogenised in Hank’s balanced salt solution and the cellular debris cleared by low-speed centrifugation at about 3,000 to 4,000 *x g*. The homogenates were filtered with a 45 μm pore size and the clarified homogenate inoculated onto monolayers of African Green Monkey kidney (Vero) cells (ATCC-CCL81). The infection was carried out at 37°C and infected cells were observed for cytopathic cell effect (CPE) under a light microscope. The viruses isolated from the heart and lung tissues were designated as Sing-M105-02 and Sing-M100-02, respectively. Sing-M105-02, Sing-M100-02 and EMCV(Aust) were propagated in Vero cells in minimal Eagle’s medium (MEM) (Invitrogen) supplemented with 5% fetal calf serum (FCS) (Hyclone, Logan) at 37°C, 5% CO_2_ for 3 days. The culture supernatant was then harvested, centrifuged at 4,000 *x g* and stored at -80°C. EMCV(Aust) strain was imported into Singapore from Dr Peter Kirkland, MacArthur Institute, Australia. The EMCV(Aust) strain was isolated from a rodent in an Australian zoo as reported in Reddacliff *et al.*, [3]. Virus isolates and EMCV(Aust) were also inoculated into Mardin Darby Canine Kidney (MDCK) cells (ATCC-CCL34) and CPE observed over a few days.

### Virus neutralization

The antiserum was serially diluted in two-folds and mixed with a known titer of either Sing-M105-02 or EMCV(Aust) in equal volume for 1 hr at 37°C. The antiserum-virus mixtures were next inoculated onto Vero cells seeded in a 96 well formant and examined daily for signs or absence of CPE.

### Electron microscopy

Virus-infected tissue culture fluid was clarified by low-speed centrifugation at 2,000 to 3,000 *x g* for 10 min. The virus was pelleted from the supernatant by ultracentrifugation at 159,000 *x g* for 1.5 h at 4°C and resuspended in PBS [20]. Drops of virus suspension were placed on carbon-formvar coated grids. Negative staining was performed with 2% phosphotungstic acid, pH 7.4, for 1 min. Grids were examined with a JEOL-1010 electron microscope.

### Immunofluorescence assay

Virus-infected cells were scraped off from a 25 cm^2^ tissue culture flask at 24 h pi and washed in phosphate-buffered saline (PBS), pH 7.4, by low speed centrifugation at 2,000 to 3,000 *x g* for 10 min. The cell pellet was resuspended in 1 ml of PBS and 5 μl of cell suspension was distributed into each well of a Teflon-coated slide and air-dried. Cells were fixed in cold acetone for 10 min and virus antigens detected by immunofluorescence using polyclonal anti-EMCV raised in pig as the primary antibody (a gift from Dr Peter Kirkland, MacArthur Institute, Australia). The secondary antibody was goat anti-porcine IgG, conjugated to fluorescein isothiocynate (Chemicon). Evans Blue (Sigma Diagnostics) was used as counter-stain and the slide examined with a Olympus DX51 microscope.

### Primers for virus identification and sequencing

For the initial determination of the virus genus and species, we used a total of 4 sets of PCR primers. Two published primer sets, RotbartFor/RotbartRev and EV1/EV2, both for the detection of enterovirus [24,25], and another two primer sets, P9/P10 (targeting the capsid VP3/VP1 gene) and P1/P2 (targeting the 3Dpol gene), for the detection of EMCV were selected [13]. The sequences and references of these primers sets are described in Table 1. The primers used for subsequent full genome assembly and sequencing were designed by primer walking, and are shown in Table 2.

### RNA extraction, cDNA synthesis and PCR

Viral RNA was extracted from 100 μl of virus-infected Vero cell culture supernatant using the RNeasy Mini Kit (Qiagen) as per the manufacturer’s instructions. RNA was eluted in 50 μl of RNase-free water. For initial virus identification, the first strand cDNA was synthesized with gene-specific primers (2 μM) (Table 1) using the Superscript III First Strand Synthesis System for RT-PCR (Invitrogen, USA) as per the manufacturer’s protocol. For virus genome amplification and sequencing, the first strand cDNA was synthesized using using either random hexamers (50 ng/ μl) or gene-specific primers (2 μM) (Table 2) using the Superscript III First Strand Synthesis System for RT-PCR (Invitrogen, USA) as per the manufacturer’s protocol.

For PCR, 2 μl of each viral cDNA sample was amplified in a 50 μl reaction mixture (33.5 μl RNase-free water, 5 μl 10x High Fidelity (HiFi) PCR Buffer, 5 μl 2 μM dNTPs, 2 μl 50 mM MgSO_4_, 1 μl 10 μM forward primer, 1 μl 10 μM reverse primer, 0.5 μl 50 U/μl HiFi Platinum Taq). Thermal cycling conditions included initial denaturation at 95°C for 5 min; followed by 45 cycles of 95°C for 30 s, 45°C for 30 s, 68°C for 2 min; and a final extension step of 68°C for 10 min. Touchdown PCR was also performed in several instances to improve PCR amplification specificity.

To obtain the 3′ end sequence of the virus, the GeneRacer kit and core module primers (Invitrogen, USA) were employed as per the manufacturer’s instructions. We were unable to obtain about 200 nt of the virus 5′ UTR sequence despite numerous attempts using the GeneRacer kit, other reverse transcriptases, DNA polymerases, PCR additives (such as DMSO and betaine), and various PCR thermocycling conditions.

### Sequencing and phylogenetic analysis

PCR products were resolved on 1-2% agarose gels (depending on amplicon size), and the subsequent PCR clean-up and sequencing of the amplicons were performed by AITbiotech (Singapore) using BigDye chemistry (Life Technologies, USA). In brief, the genomes of EMCV isolates Sing-M100-02 and Sing-M105-02 were determined from a series of PCR amplicons that contained overlapping cDNA fragments. Each gene region was sequenced at least two times. Raw sequences were first assembled and aligned with the reference EMCV Mengo M virus genome (GenBank accession number L22089) using the SeqMan Pro software found in the Lasergene Version 8 (DNASTAR, USA).

Final nt sequences were aligned against other strains of EMCV and picornaviruses using the Clustal W method as implemented in the MegAlign program (DNASTAR, USA). Percent identity and phylogenetic trees were constructed for the entire ORF as well as for individual genes coding for the various proteins at the nt level and neighbor-joining trees were constructed after alignment by the same program. The consistency and robustness of tree topology were bootstrapped using 1000 trials and a seed of 111. The TreeView X 1.6.6 program [50] was used to visualize trees.

### Nucleotide sequence accession numbers

The partial sequence data for the VP3/VP1 genes have been deposited in GenBank under the accession numbers AF525466 for Sing-M105-02 and AY162280 for Sing-M100-02. For the partial 3Dpol genes, the nt sequences were deposited under the accession numbers AF510055 for Sing-M105-02, and AY162279 for Sing-M100-02. The full virus genome sequences (excluding about 200 nt from the 5′ end) have been deposited in GenBank under accession numbers KC310737 for Sing-M100-02, and KC310738 for Sing-M105-02.

## Competing interests

The authors declare that they have no competing interests.

## Authors’ contributions

DS-YY, JEL, JCWL, EASL and HCT performed the laboratory experiments. SO performed the clinical assessment. CJF performed the necropsy and sampling. CJF, Y-NL and M-LS performed the virus isolation. B-HT, M-LN, E-EO, K-PC, SO and CJF conceived the study. B-HT and DS-YY were involved in the data analysis and manuscript writing. All authors read and approved the final manuscript.
